# Comparative Evaluation of Ovsynch and Double Ovsynch Protocols with Single and Double Insemination in Holstein Dairy Cows: Reproductive Performance and Cost Analysis

**DOI:** 10.3390/ani15162380

**Published:** 2025-08-13

**Authors:** Daniel Ionut Berean, Liviu Marian Bogdan, Raluca Cimpean

**Affiliations:** 1 Department of Reproduction, Faculty of Veterinary Medicine, University of Agricultural Sciences and Veterinary Medicine Cluj-Napoca, Calea Manastur 3–5, 400372 Cluj-Napoca, Romania; liviu.bogdan@usamvcluj.ro; 2 Department of Animal Breeding and Food Safety, Faculty of Veterinary Medicine, University of Agricultural Sciences and Veterinary Medicine Cluj-Napoca, Calea Manastur 3–5, 400372 Cluj-Napoca, Romania; calina-raluca.cimpean@usamvcluj.ro

**Keywords:** Double Ovsynch, estrus synchronization, artificial insemination, dairy cow reproduction, pregnancy rate, economic efficiency

## Abstract

This study investigated the reproductive and economic costs of four estrus synchronization protocols in multiparous Holstein dairy cows: Ovsynch with one or two artificial inseminations (AIs), and Double Ovsynch with one or two AIs. A total of 216 clinically healthy cows were randomly assigned to one of the four treatment groups (n = 54 each). Pregnancy diagnosis was performed by transrectal ultrasonography at 33 days post insemination. Economic calculations included the cost of hormonal treatments, insemination, and pregnancy diagnosis, reflecting market prices in large scale Romanian dairy operations. The highest pregnancy rate (64.8%) and lowest cost per confirmed pregnancy (EUR 89.51) were achieved in the group treated with Double Ovsynch followed by a single AI. The standard Ovsynch protocol with one AI yielded the lowest conception rate (42.6%), indicating limited effectiveness under fixed-time AI conditions. These findings suggest that Double Ovsynch with a single insemination is the most effective and economically viable protocol for reproductive management in intensively managed dairy herds.

## Simple Summary

Improving fertility in dairy cows is important for both farm productivity and profitability. This study looked at two hormone-based programs used to control when cows ovulate: Ovsynch and Double Ovsynch. Each program was tested with one or two inseminations to see which combination worked best. The study was performed on a large dairy farm in Romania, using Holstein cows in good health. The results showed that Double Ovsynch with only one insemination gave the best pregnancy rate and the lowest cost per pregnancy. These findings offer practical advice for veterinarians and dairy producers looking to choose the most effective and affordable reproductive strategy for their herds.

## 1. Introduction

In recent decades, reproductive biotechnologies have become essential tools in the management of dairy cattle herds. The continuous demand for high milk yield and reproductive efficiency in commercial dairy farms has led to the implementation of hormonal protocols that facilitate the synchronization and induction of estrus [1]. One of the major challenges in modern dairy herd management is the accurate detection of estrus, a critical factor influencing reproductive success. In high-producing dairy cows, estrus expression is often silent or markedly shortened due to factors such as metabolic stress, negative energy balance, and subclinical disease [2,3,4]. Visual observation of estrus is labor-intensive and often unreliable, particularly in large-scale operations. These limitations lead to missed breeding opportunities, extended calving intervals, and reduced herd productivity. Consequently, the cattle industry increasingly adopts synchronization protocols to bypass the need for estrus detection and improve the timing of artificial insemination, thus enabling more consistent and efficient reproductive management [1,4].

Reproductive efficiency plays a critical role in the economic sustainability of dairy operations. Maintaining a consistent calving interval, ideally one calf per cow per year, is essential to support continuous milk production and effective herd turnover. To achieve this, advanced reproductive strategies must be employed, particularly in large-scale, high-performance systems where precision and predictability are crucial [5,6,7]. Among the most commonly used protocols worldwide are Ovsynch and Double Ovsynch, designed to optimize the reproductive performance of dairy cows by allowing fixed-time artificial insemination (FTAI) without the need for estrus detection [8,9].

The Ovsynch protocol, based on the sequential administration of Gonadotropin-Releasing Hormone (GnRH) and Prostaglandin F2 alpha (PGF_2_α), has proven effective in synchronizing follicular waves, inducing luteolysis, and triggering ovulation, thereby enabling FTAI at a precise time [10,11]. The Double Ovsynch protocol, which includes a preparatory hormonal sequence before the standard Ovsynch, has been shown to enhance conception rates by optimizing ovarian status and uterine conditions before insemination [12,13].

In addition to the timing of ovulation, the number of inseminations per estrous cycle has been explored as a method to improve conception rates. Double artificial insemination, performed approximately 12 h apart, is based on the premise of extending sperm availability during the fertile window and increasing the likelihood of fertilization. While some studies suggest that double insemination can improve conception in cases of uncertain estrus timing or delayed ovulation, other research indicates minimal benefit when precise synchronization protocols are used. In protocols like Ovsynch, where ovulation is tightly controlled hormonally, the added value of a second insemination remains controversial. However, in field conditions where metabolic stress, individual variability in ovulatory response, or technical inconsistencies may influence outcomes, double insemination is still widely applied in practice. Evaluating its efficacy under controlled synchronization protocols remains relevant for optimizing reproductive efficiency and cost-effectiveness in commercial dairy herds [14,15,16,17,18].

The aim of this study was to evaluate and compare the reproductive performance and economic efficiency of two commonly used hormonal synchronization protocols, Ovsynch and Double Ovsynch, in multiparous Holstein dairy cows managed under intensive commercial conditions. Additionally, the study examined the effect of performing a single versus a double AI within each protocol, resulting in four treatment groups. It was hypothesized that the Double Ovsynch protocol combined with a single AI would provide the highest pregnancy rate and the lowest cost per confirmed pregnancy, due to improved synchronization of ovulation and more efficient use of reproductive resources. By analyzing both pregnancy outcomes and cost per confirmed pregnancy, the study seeks to provide a comprehensive understanding of how protocol selection and insemination strategy influence fertility and profitability.

## 2. Materials and Methods

### 2.1. Study Location and Duration

The study was carried out between October 2023 and May 2024 at a large commercial dairy farm located in Alba County, Romania (45.9322° N, 23.5897° E). The farm operates at an intensive production level and is among the largest integrated livestock facilities in the region, both in terms of herd size and technological infrastructure. The farm houses over 1800 dairy cows, primarily of the Holstein Friesian breed, managed under a high-input, high-output system. The facility includes a rotary milking parlor with 60 stalls, capable of milking 300–350 cows per hour, and produces approximately 40,000 L of milk daily. Milk is rapidly cooled from 37 °C to 4 °C using an industrial cooling system and is directly loaded into transport tankers to preserve hygiene and quality.

The farm is subdivided into multiple specialized units and animal groups, including lactating cows, dry cows, postpartum cows, heifers, and calves. Each category is housed separately in semi-open barns, allowing free access to feed, water, and resting areas. Animal comfort and hygiene are carefully managed, with regular manure removal and tailored bedding systems that vary seasonally (e.g., straw mixed with lime during winter and sand during summer).

Nutrition is delivered through a Total Mixed Ration (TMR) system using a technologically advanced mixing wagon. Rations are carefully formulated based on the animal’s physiological stage, with variations for high yielding cows, transition cows, dry cows, and growing heifers. Information regarding the ration used on the farm is provided in the Supplementary Materials (Table S1). The TMR for lactating cows includes approximately 47 kg of feed per cow per day, composed of silage maize, alfalfa silage, triticale, straw, brewer’s yeast, and a custom concentrate mix. The farm also integrates by products such as brewer’s yeast to enhance rumen fermentation and feed efficiency. Water and feed access are unrestricted, and feed bunk management is routinely monitored to ensure intake consistency.

Animal health, reproduction, and productivity are continuously monitored by an in-house veterinary team, supported by trained technicians and automated data systems. The farm’s infrastructure and management practices provided a controlled environment suitable for evaluating the reproductive protocols applied in this study.

### 2.2. Animal Selection and Experimental Design

This study was conducted on 216 multiparous Holstein-Friesian dairy cows reared under uniform management, housing, and nutritional conditions. Animals were enrolled between 40 and 60 days postpartum, having completed uterine involution and being clinically healthy at the time of inclusion. Only cows with a Body Condition Score (BCS) between 2.5 and 3.5 (on a 5-point scale) were selected. No reproductive disorders, systemic illness, or recent antibiotic treatments were recorded (Table 1). The cows were randomly allocated, regardless of previous parity or lactation performance, into four experimental groups (n = 54 each), designed to compare different synchronization and insemination strategies.

The groups were defined as follows:

- Group OV1: Ovsynch protocol followed by a single fixed-time artificial insemination (FTAI).
- Group OV2: Ovsynch protocol followed by two inseminations, 12 h apart.
- Group DOV1: Double Ovsynch protocol followed by a single FTAI.
- Group DOV2: Double Ovsynch protocol followed by two inseminations, 12 h apart (Table 1).

**Figure 1:**
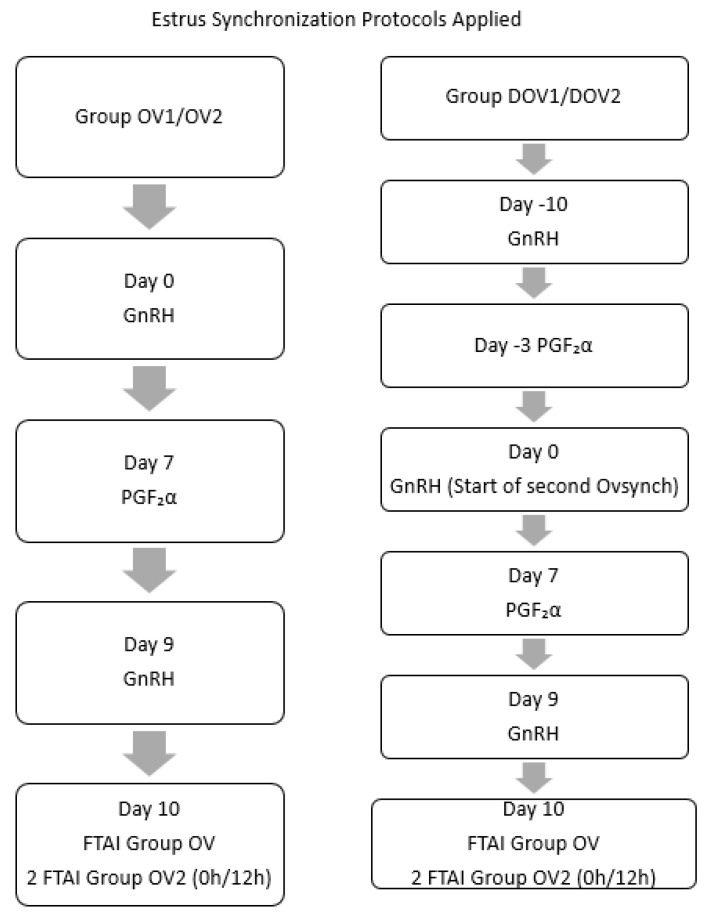
Estrus synchronization protocols applied in the four experimental groups. All hormonal treatments were administered intramuscularly. Double Ovsynch protocols included a pre-synchronization phase to enhance ovulatory response. FTAI = fixed-Time artificial insemination; OV1 = Ovsynch Group (single AI); OV2—Ovsynch Group (double AI at 0h/12h); DOV1—Double Ovsynch Group 1 (single AI); DOV2—Double Ovsynch Group 2 (double AI at 0 h/12 h); PGF_2_α—Prostaglandin F2 alpha; GnRH—Gonadotropin-Releasing Hormone.

All hormonal treatments used in the synchronization protocols were administered intramuscularly by a veterinarian. Two commercial products were employed: Receptal (buserelin acetate, 0.0042 mg/mL, Maravet, Baia Mare, Romania) as a GnRH analog (2.5 mL/animal/administration) and Estrumate (cloprostenol, 0.25 mg/mL, Maravet, Baia Mare, Romania) as a PGF_2_α analog (2 mL/animal/administration). The synchronization protocols were applied in two main experimental groups: Group OV1/OV2 and Group DOV1/DOV2, as illustrated in Figure 1.

For Group OV1/OV2, the protocol commenced on Day 0 with an intramuscular administration of buserelin acetate. On Day 7, animals received cloprostenol, followed by a second buserelin acetate injection on Day 9. FTAI was performed on Day 10, with Group OV animals inseminated once and Group OV2 animals inseminated twice, at 0 and 12 h.

For Group DOV1/DOV2, a Double Ovsynch protocol was implemented. The first buserelin acetate injection was administered on Day −10. Seven days later, on Day −3, cloprostenol was given. On Day 0, animals received a second buserelin acetate injection, marking the start of the second Ovsynch sequence. A subsequent cloprostenol injection was administered on Day 7, followed by a final buserelin acetate injection on Day 9. FTAI was performed on Day 10, following the same schedule as in Group OV/OV2: one insemination at 0 h for Group DOV1 and two inseminations at 0 and 12 h for Group DOV2.

In all groups, sexed Holstein semen from the same high genetic merit sire was used to eliminate variability in male reproductive performance. Inseminations were performed by a single trained technician using the recto vaginal technique to ensure consistency.

Pregnancy diagnosis was carried out on Day 33 post insemination using transrectal ultrasonography (Easy Scan Go, Maravet, Baia Mare, Romania). Animals that returned to estrus within 21 days of insemination were recorded as non-pregnant and excluded from further reproductive interventions during the study period.

### 2.3. Economic Evaluation

An economic analysis was performed to compare the cost efficiency of the four estrus synchronization and insemination protocols applied in this study. The assessment was based on the calculation of the cost per confirmed pregnancy in each group, using the following formula:

Cost per pregnancy = Total cost per group/Number of pregnancies confirmed at Day 33

The total cost per group was calculated by summing all expenditures related to hormonal treatments, artificial insemination procedures, and pregnancy diagnosis. The following unit prices were used, reflecting average market values commonly applied in large commercial dairy farms in Romania, where veterinary services are contracted at scale (typically involving visits for more than 50 animals at a time):

- GnRH administration (Receptal): 4 EUR/dose.
- PGF_2_α administration (Estrumate): 3 EUR/dose.
- Artificial insemination (sexed semen + labor): 20 EUR/insemination.
- Pregnancy diagnosis (transrectal ultrasonography): 4 EUR/examination.

All prices include both the cost of pharmaceuticals and the standard veterinary labor fees under routine commercial practice. Calculations were made independently for each experimental group, allowing comparison of reproductive efficiency not only in biological terms (pregnancy rate), but also from an economic perspective relevant to decision-making in herd reproductive management.

### 2.4. Statistical Analysis

Data were analyzed using the Statistical Package for the Social Sciences (SPSS), version 25.0 (IBM Corp., Armonk, NY, USA). The primary outcome variable was pregnancy rate, defined as the proportion of cows diagnosed pregnant at 33 days post insemination out of the total number of cows inseminated per group.

Differences in pregnancy rates between experimental groups were evaluated using the Chi-square (χ^2^) test for independence, with pairwise comparisons adjusted using the Bonferroni correction to control for multiple testing. The threshold for statistical significance was set at *p* < 0.05.

Cost per pregnancy was calculated descriptively for each group and expressed as mean cost per confirmed pregnancy (EUR). Because economic data are ratio level and not normally distributed, no inferential statistics were applied to cost comparisons; instead, results were interpreted based on relative cost effectiveness.

All data were reported as percentages, means, and total values, and presented in tabular form. Graphical representations were used where appropriate to illustrate differences in economic outcomes across groups.

## 3. Results

### 3.1. Pregnancy Results

At 33 days post insemination, differences in pregnancy rates were observed among the four experimental groups (Table 2). The highest pregnancy rate was recorded in Group DOV1, with 64.8% (35/54) of cows confirmed pregnant. Group DOV2 showed a pregnancy rate of 61.1% (33/54), with no statistically significant difference compared to Group DOV1 (*p* > 0.05).

Group OV1 had a pregnancy rate of 42.6% (23/54), which was significantly lower than Group DOV1 (*p* < 0.05). Group OV2 achieved a pregnancy rate of 50.0% (27/54), with no significant difference compared to the other groups.

### 3.2. Economic Results

**Figure 2:**
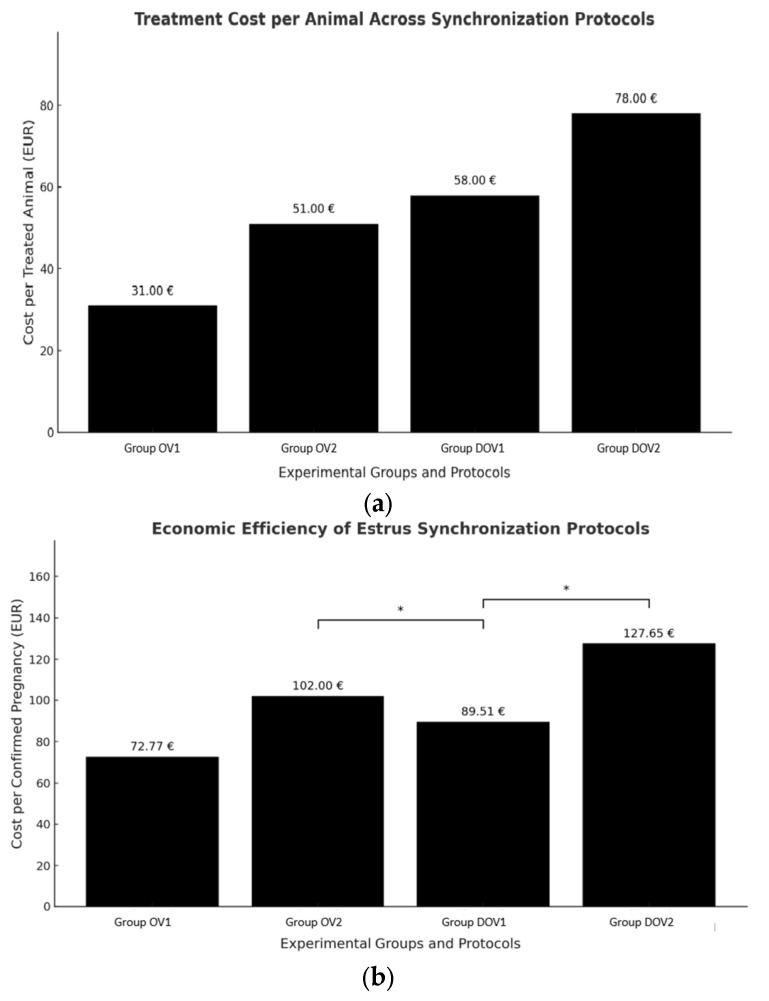
Economic results. (**a**) Average treatment cost per cow for each estrus synchronization protocol. Values include the cost of GnRH and PGF_2_α administration, artificial insemination, and pregnancy diagnosis. These reflect typical veterinary service pricing applied in Romanian large-scale dairy operations (≥50 animals per visit); (**b**) cost per confirmed pregnancy calculated as total reproductive cost per group divided by the number of pregnancies at Day 33. Asterisks (*) denote statistically significant differences (*p* < 0.05) compared to Group DOV1 (Double Ovsynch + 1 AI), which was the most cost-effective protocol.

The economic analysis revealed notable differences in cost efficiency among the four estrus synchronization protocols. The average cost per treated animal ranged from 31.00 EUR in Group OV1 to EUR 78.00 in DOV2 (Figure 2a). Group DOV1 demonstrated the most favorable outcome, with a pregnancy rate of 64.8% and a cost per confirmed pregnancy of only EUR 89.51, representing the best cost effectiveness ratio observed. In contrast, Group DOV2 incurred the highest cost per pregnancy at EUR 127.65, suggesting that the addition of a second insemination did not yield sufficient benefit to justify the extra expense. Among the Ovsynch based groups, Group OV2 achieved a pregnancy rate of 50.0% but at a cost of EUR 102.00 per pregnancy, substantially higher than OV1, which, although having a lower pregnancy rate of 42.6%, maintained a cost per pregnancy of EUR 72.77 (Figure 2b).

## 4. Discussion

Synchronization protocols play a crucial role in improving reproductive efficiency in dairy herds by enhancing the timing and success of artificial insemination. Their impact on pregnancy rates is significant, particularly in high producing cows where fertility tends to be compromised. In this study, a comprehensive comparison of four estrus synchronization and insemination protocols was conducted, assessing both reproductive performance and economic efficiency. The results demonstrate that the Double Ovsynch protocol, especially when used with a single, well-timed insemination, offers the greatest reproductive advantage. Group DOV1, which followed this approach, achieved the highest pregnancy rate (64.8%) and the lowest cost per confirmed pregnancy (EUR 89.51), identifying it as the most efficient strategy based on both biological and economic outcomes.

When comparing Group DOV1 to Group OV1, the advantages are striking. Group OV1 achieved the lowest pregnancy rate (42.6%), which was significantly lower than that of Group DOV1. This finding is consistent with earlier studies indicating that the standard Ovsynch protocol, while widely used, often suffers from suboptimal outcomes when implemented in high yielding cows during early lactation [19,20]. This reduced efficiency may be attributed to asynchronous follicular waves, subclinical uterine health issues, and inadequate luteolysis at the time of insemination, factors that the Double Ovsynch protocol is designed to address. By pre-synchronizing the ovarian status before initiating the standard Ovsynch sequence, Double Ovsynch improves ovulatory response consistency and prepares the reproductive tract for a more successful conception [5,12,21].

Although Group DOV2 achieved a relatively high pregnancy rate (61.1%), the difference compared to Group DOV1 was not statistically significant. However, the additional insemination significantly increased the cost per pregnancy (EUR 127.65), representing the least cost-effective approach in the study. This observation aligns with recent studies by Consentini et al. [22] and López-Gatius [23], which have demonstrated that additional inseminations within an FTAI framework rarely improve fertility when ovulation timing is tightly synchronized. Sperm viability and uterine receptivity are already optimized by the hormonal protocol; therefore, a second insemination may introduce unnecessary cost without a corresponding increase in pregnancy probability.

In Group OV2, the strategy of administering two inseminations did improve the pregnancy rate to 50.0%, an 8% increase over Group OV1. However, this improvement did not reach statistical significance when compared to Group DOV1, and the cost per pregnancy rose substantially to EUR 102.00. This suggests that in the absence of pre-synchronization, increasing the number of inseminations alone is an inefficient compensatory mechanism for reproductive timing variability. These findings echo those of Carvalho et al. [24], who argued that while double AI may slightly improve pregnancy rates in cows with ambiguous estrus expression or delayed ovulation, it remains biologically inferior and economically unjustified in the context of FTAI protocols where ovulation is exogenously controlled.

When evaluating Group OV1 vs. Group OV2, the data confirm the expected but modest benefit of double insemination in a standard Ovsynch protocol. However, this improvement comes at a 65% increase in cost per pregnancy (from EUR 72.77 to EUR 102.00), indicating a poor return on investment. Thus, from a herd management standpoint, double insemination may not be advisable unless supported by individual reproductive history or ovulatory diagnostics, as also suggested by Fricke and Wiltbank [25].

The economic implications of these findings are particularly relevant for large-scale commercial dairy farms, where even small improvements in reproductive efficiency can result in substantial gains at the herd level [26]. The pricing model used in this study reflects real world conditions in Romanian dairy systems, where veterinary services are standardized and often applied to groups of ≥50 animals per visit. Under such conditions, the ability to maximize pregnancy rates with minimal hormonal and labor inputs becomes critical for operational sustainability. The Double Ovsynch + 1 AI protocol clearly meets these criteria and is thus strongly recommended for reproductive management in herds with similar structure and performance metrics.

Furthermore, our findings reinforce a growing consensus in the literature that pre-synchronization strategies are essential to overcome the limitations of early postpartum anestrus, silent heat, and metabolic stress in high-producing Holstein cows [27,28]. The advantages of Double Ovsynch have been confirmed across various geographic and management contexts, including studies in North America [9], Europe [29], and Latin America [30], underscoring its adaptability and robustness.

While this study offers valuable evidence supporting the reproductive and economic advantages of the Double Ovsynch protocol, it is important to consider some context when interpreting the results. The research was conducted in a single high-performance commercial dairy herd under specific Romanian management and nutritional conditions, which may mean the findings are most directly applicable to similar herds. Herds with different structures, breeds, climates, or reproductive histories might experience different outcomes. Our study focused exclusively on clinically healthy, multiparous Holstein cows with moderate body condition scores (3.0–3.5). This selection was intentional, as including primiparous, underconditioned, or metabolically challenged animals might have introduced additional variability, especially considering that conditions such as anestrus are more prevalent in these groups [31]. Nevertheless, future research including these categories would be valuable to assess the protocol’s broader applicability.

Furthermore, the hormonal protocols were evaluated without endocrinological monitoring (e.g., progesterone assays) or ultrasonographic tracking of follicular dynamics, as the study was designed to closely reflect practical, clinical conditions encountered in commercial herds. However, incorporating such monitoring in subsequent investigations could provide deeper physiological insights and enhance understanding of the protocol’s mechanisms. Comparative studies involving sexed versus conventional semen, metabolic fertility indicators, and long-term productivity metrics, including calving interval, days open, and culling rate, will also be essential to expand the clinical relevance of these protocols. Moreover, multi-site and cross-country economic evaluations could help validate the financial feasibility of these strategies across diverse production systems and veterinary service models.

## 5. Conclusions

The Double Ovsynch protocol followed by a single timed AI achieved the highest pregnancy rate (64.8%) and the lowest cost per confirmed pregnancy (89.51 EUR), demonstrating superior reproductive and economic efficiency. This protocol is a practical and cost-effective strategy for fertility management in intensive dairy herds.

## Figures and Tables

**Table 1:** Descriptive statistics of cows by treatment group (mean ± SD).

Group	Average BCS (Mean ± SD)	Average DIM (Mean ± SD)	Average Parity (Mean ± SD)
OV1	3.18 ± 0.15	52.6 ± 4.9	2.7 ± 0.5
OV2	3.24 ± 0.17	53.8 ± 4.6	2.9 ± 0.4
DOV1	3.16 ± 0.14	51.7 ± 4.3	2.8 ± 0.5
DOV2	3.23 ± 0.16	54.1 ± 4.7	2.9 ±0.5

BCS = Body Condition Score; DIM = Days in Milk.

**Table 2:** Pregnancy rates at 33 days post insemination across experimental groups.

Group	Protocol	Pregnant Cows (n/54)	Pregnancy Rate (%)
Group OV1	Ovsynch + 1 AI	23/54	42.6% *
Group OV2	Ovsynch + 2 AI	27/54	50.0% ^ns^
Group DOV1	Double Ovsynch + 1 AI	35/54	64.8%
Group DOV2	Double Ovsynch + 2 AI	33/54	61.1% ^ns^

Group DOV1 showed a significantly higher pregnancy rate compared to OV1 (*p* < 0.05). Differences between DOV1 and Groups OV2 and DOV2 were not statistically significant. Asterisk (*) indicates significantly lower pregnancy rate compared to Group DOV1. “^ns^” denotes no statistically significant difference.

## Data Availability

Data are contained within the article.

## Supplementary Materials

The following supporting information can be downloaded at: https://www.mdpi.com/article/10.3390/ani15162380/s1, Table S1: Total Mixed Ration (TMR) Composition for Different Cattle Categories (kg/animal/day).
