# Supplementary material for: Comparative Evaluation of Ovsynch and Double Ovsynch Protocols with Single and Double Insemination in Holstein Dairy Cows: Reproductive Performance and Cost Analysis
Source: Animals (Basel). 2025 Aug 13;15(16):2380. doi: 10.3390/ani15162380 (PMC12383117; doi:10.3390/ani15162380)
Supplement: Supplementary file 1 [file animals-15-02380-s001.zip › animals-3768490-supplementary.pdf]

Table S1. Total Mixed Ration (TMR) Composition for Different Cattle Categories (kg/animal/day).

Feed Component*	Lactating Cows	Fresh Cows	Dry Cows	Transition Cows	Heifers
Straw	1.0	0.5	4.5	1.0	–
Alfalfa silage	10.0	14.0	5.0	5.0	11.0
Corn silage	15.0	17.0	8.0	14.0	11.0
Triticale	5.0	–	–	–	–
Concentrate mix (grain + premix)	13.0	6.0	–	3.0	–
Brewer's yeast	3.0	3.0	–	–	–
Sunflower meal	–	–	1.0	–	0.5
Corn meal	–	–	–	–	1.5
Minerals	–	–	0.15	–	0.15
Salt	–	–	0.10	–	0.10
Total (kg/day)	47.0	40.5	18.75	23.0	24.25

*Ration formulations are based on the physiological stage and production level of each group. All diets are formulated to meet the nutrient requirements for dairy cattle. Ingredient availability and farm-specific nutritional strategies, including the use of brewer's yeast as a rumen stimulant and triticale as an energy source, were taken into account. Feed was delivered via a mixing wagon to ensure uniformity and consistent intake.
